# Pensions for Hospital Employees

**Published:** 1914-07-04

**Authors:** P. J. Michelli

**Affiliations:** C.M.G.; Secretary Seamen's Hospital Society, Greenwich, and the London Schools of Tropical and Clinical Medicine


### PENSIONS FOR HOSPITAL EMPLOYEES.

**By P. J. MICHELLI, C.M.G., Secretary Seamen's Hospital Society, Greenwich, and the London Schools of Tropical and Clinical Medicine.**

The question of pensions for hospital officials has occupied the attention of boards of management for a very long time, but until this year there has not appeared any reasonable prospect of a solution of the problem as to whether an old and worn-out official is or is not entitled to a pension.

Up to the present the boards of management of most hospitals have approached the subject in an uncertain and perplexed state of mind, yet it must be admitted that few faithful servants who have spent the best years of their lives in the interest of the sick and suffering have been allowed to leave unrewarded when by reason of age they can no longer carry out the duties of their office.

On the whole the old employee is given an adequate retiring allowance; at the present time practically every hospital employee of long service is given a pension on a non-contributory basis, but there are many drawbacks to the lack of system that prevails. Not the least is the inequality of the grants made by different boards, so that similar services meet with greatly varying rewards. Quite recently two senior officers in large London hospitals have been retired, one on £350 per annum and the other on 30s. a week, though both hospitals are practically of the same size and the status, the duties, and length of service of the officials were the same.

Such a difference as this is not due to the lack of a desire on the part of the board to deal fairly by their employee. It arises, as I have said, from a genuine doubt as to whether a pension is properly and rightly due to the retiring official and from the complete uncertainty as to what should be its amount.

The different scale upon which the pension is given is also due to the changing nature of any board; while as the employee has no legal claim he has perforce to submit to the decision of those members who may chance to attend on the day his case is considered.

No guarantee of a pension is given when the employee enters the service of the hospital, and the board admits no liability. In these circumstances the superannuation is granted in the light of a benevolence, and the recipient must take it or leave it and be thankful.

The decision of a board also fluctuates according to the financial position of the hospital at the moment that the question is under consideration. If the hospital happens to be having a good year the retiring official comes off all right, but if there happens to be a deficiency, or there is an overdraft at the bank, he is not likely to fare well. Nothing could be more inconsistent and indeed unfair than this.

Perhaps the greatest hardship of such a *régime* is the painful anxiety of the old official who feels his powers failing and yet is fearful to submit his case to the unknown fate that awaits him.

Thus the present state of affairs calls for action from some authoritative body; boards of management are willing enough to grant pensions, but the two essential conditions of certainty and uniformity have not yet been secured. Fifty years ago the committee of the Middlesex Hospital passed resolutions approving of pensions to salaried officers, sisters, nurses, and servants on a definite scale; at about the same time the London Hospital took similar action in regard to the nursing staff; but the first indication that pensions had received what may be called official sanction was when the great central Funds allotted a place in their returns for an entry of the amount annually expended upon pensions, thus admitting pensions as a legitimate expenditure, and when the Secretary of the Sunday Fund was granted a pension. This was a distinct step in advance, and gave confidence to many boards of management when dealing with the subject, but there was no authoritative guide as to what the rate of pension should be.

Towards the closing years of the last century came the pension funds of Guy's, the London, and St. Bartholomew's. These three pioneers compiled complete schemes, practically on the lines of the Superannuation Act of 1859, 22 Vict., cap. 26.

It may be here convenient to give a short summary of that Act, which is the basis upon which every employee under the Crown is pensioned. It is as follows :—

One-sixtieth of maximum pay and emoluments for each year of service, with the following conditions :—

No pension under ten years' service.

No period beyond forty years' service to count for pension, and retirement at not less than sixty years of age, unless incapacitated.

The Act also gives reasonable discretion for rewarding meritorious service, for it is enacted in Section IV. that any number of years not exceeding twenty may be added to the actual number of years' service upon which the amount of the pension is calculated.

In the case of the above-mentioned hospitals no contribution is exacted from the employee, and no material amount is set aside for the purpose of a pension fund or to provide a reserve. All have regarded the incidence of pensions as a legitimate disbursement in the annual cost of maintenance. In fact, they have regarded this item on the expenditure side of the account as an uncertain and variable charge, much in the same way as legacies are regarded as an uncertain and variable source of income. Both are items that vary from time to time, but which, as Lord Knutsford has shown, in the case of legacies may be averaged over a series of years with much accuracy. Indeed, expenditure on pensions may be regarded as more easily and accurately calculable than revenue derived from testamentary bequests.

Practically all charities, and especially hospitals, regard legacies, though varying from year to year, as ordinary income. The majority of hospitals could not meet their annual liabilities without them, and they are invariably taken into account, though some hospitals go through the formality of crediting them to some fanciful account, or to the balance sheet, before bringing them into the income and expenditure account. The two items, one of income and the other of expenditure, have some phases in common, and may reasonably be treated alike.

Since the hospitals I have referred to adopted their pension schemes they have been followed by others on similar lines. Among these may be mentioned the Cancer, the Ventnor Hospital for Consumption, the Manchester Royal Infirmary, and the Poplar Hospital. Now in this year of grace King Edward's Hospital Fund for London has announced, in response to a request from the Incorporated Association of Hospital Officers, that it is prepared to institute an inquiry into the subject, and from the nature of the reference which the executive of the Fund has made to a special committee it is clear that the Fund is about to approach the matter in a thorough and impartial manner, and employees in London may look forward with confidence to the issue of their report.

### Pensions as Sound Policy.

There are other and weightier reasons why this question of pensions should be dealt with than the feelings and claims of hospital employees, and doubtless the King's Fund has these in mind.

The business of managing a hospital is one that demands high qualities of organisation—just those qualities for which the commercial world and the State pay the highest price. There is no reason why any man should give these services to the voluntary hospitals either free or at a lower rate than to the Civil Service, the Army, or the Navy. It is bad policy to offer low remuneration in hospital life. Occasionally a man may for special reasons work for a small monetary return, but in the long run the voluntary hospitals will get the officials they pay for and nothing better.

The future of the hospitals greatly depends upon efficient management, and it is only by securing the best men that this can be assured. It must therefore be to the interest of the hospitals to attract able and suitable employees.

There can be no doubt that this uncertainty as to the future has deterred many men from adopting hospital

**(Continued on page 386.)**

### The British Hospitals Association.

### PENSIONS FOR HOSPITAL EMPLOYEES—(continued from page 378).

life as a calling. The actual pay can never be large, never more than will enable a man to live decently and provide for those dependent upon him in the event of premature death, while it is impossible for him at the same time to provide for old age.

There is a charm about hospital life to those who know it, and they seldom leave it; but this charm is unknown to the aspirant, and consequently many good men turn aside, knowing full well that no matter how successful they may be no amount of saving can make provision for old age.

Pensions are part of the pay offered in other official walks of life, and should be a recognised certainty for the hospital official. A lower current rate of pay will attract good service if provision is made for old age. The days of easy billets in charities are past, and the public realise that hard, continuous, and responsible work is demanded of all officials, work at least as arduous and difficult as that of a Civil servant.

### The Objections to Pension Schemes.

It is only fair to set forth here the views of those who have not favoured the pensioning of officials. There seems to be an idea in the minds of some of them that the pension goes on for ever, yet it is obvious that but a small percentage of those who enter hospital employ attain pensionable age; and if that age is fixed at sixty, that the expectation of life for males is twelve years and females fourteen.

Another view is that once a certain class of official knows for certain that he will earn a pension if he lives long enough, he lapses into a mere machine doing as little as possible for his daily wage, whereas in the absence of a right to a pension he feels that he must justify by hard work his claim thereto.

This is an argument that I have heard put forward by hospital committeemen from time to time. Some of them modify their views by saying that they are "in favour of pensions for those who deserve them," but the present want of system does not ensure that result, and it deters many good men from entering hospital life, and has a discouraging effect on those who do.

The slacker we have always with us; if we attract better men to the hospitals, the fewer of these there will be.

Furthermore, it is in the interest of the hospitals that old servants should be removed and that men with youth and energy take their places. Younger men get less pay, so that the difference between the pay of the old and new hand is a set-off against the pension.

### Should they be Contributory?

Perhaps the greatest difficulty that besets the matter is to devise some scheme whereby an employee who has served in several hospitals shall be enabled to count the whole of his service towards his pension. Where the service has been continuous in one institution the matter is comparatively simple, and in practice those of this class are granted ample retiring allowances (sometimes too ample—a proposal was lately made to retire a man on full pay), but in the interest of the hospitals a man should serve in two or more institutions. It is to meet such cases that it appears necessary to establish a separate or detached pension scheme.

Many suggestions come readily to the mind, but it is not easy to formulate a scheme without coming up against many difficulties. To restrict the scheme to London is only to minimise the difficulty and to deprive the employee of the advantage of serving both in the provinces and in London.

Anything in the nature of a contributory scheme to which the employee would be required to pay any considerable proportion of the amount necessary to secure a pension would be most unpopular, and would rather deter from than attract men to hospital employment. Even proportionately raising the pay of the employee with the object of enabling him to contribute would not meet the case; preferably would a man accept a smaller salary than that generally ruling if the right to a pension were assured, so that whatever he earned he would know that he could deal with as his own and in the interests of those dependent upon him.

Besides, with a contributory scheme men look for benefits that are not in the nature of true pensions; they look for a return of a proportion of premiums paid by them in certain events, such as leaving their employ after a period of service, or in respect of benefits for wife and children in the event of premature death, etc. All such benefits are contrary to the objects for which a pension is granted, and should be provided for out of the individual's own income. Pensions must not be confounded with life assurances. The confusion of idea has been brought about by the insurance companies, who, in their endeavour to create new business, issue policies in which they combine what they call pensions or annuities with life insurance. This has led to the establishment of such schemes as the Federated Superannuation System for Universities, which is really not a pension system at all. With such schemes as these, which are provided for through the agency of insurance companies, the tendency of any man with a family is to sink his personal interest and to put as much of his money as he can away for those for whom he is responsible, and there can be little doubt that in the future the Universities will be confronted with some very awkward cases where men, after spending the whole of their lives in the service of the Universities, are left in very indigent circumstances. This appears to be a contingency which it is the duty of the employer granting the pension to guard against. The employer is not concerned with a man's wife and family; what he has to see to (and this is the spirit of all pensions) is that a servant who has served him well shall not want when he can no longer serve. Insurance for wife and family is another matter, and rests with the servant. In this it seems that the amending Act of 1909, 9 Edward 7, cap. 10, went beyond the province of a Superannuation Act.

It may be well to look at what may, and doubtless will, happen in the case of some of those provided for under the Federated Superannuation System. The bachelor or the selfish man entering the employ of a University at thirty years of age at, say, £200 per annum, will, under Option 12, retire at sixty years of age on a superannuation allowance of £131 per annum, or a cash payment, *if in health*, of £1,116, while the man with a wife and family, entering under similar circumstances and retiring at the same age, but who has taken out a policy under Option 5 or 9, must subsist on an annuity of from £57 and bonuses to £90 a year, or a cash option, *if in health*, of from £628 and bonuses to £980 without bonuses. Now, most men of sixty are not likely to pass the doctor, and therefore would not be likely to get the full cash surrender value; but should a man happen to be in good enough health to get the full surrender value, it is a doubtful benefit, for those who have spent the whole of their lives in academic pursuits or in scientific work are not generally the best judges of an investment. Many a speculation made for the first time in old age goes wrong and the victim is left without means. On the other hand, if he has a wife or other person dependent upon him, he is not likely to sink his capital in an annuity on himself. Should he be prudent he will put it out at 4 per cent. and do the best he can on less than 10s. a week; if he is imprudent, he possibly loses it all. This does not seem much like looking after a man in his old age, which is the object of all pensions.

To revert once more to Option 12 of the Universities Superannuation System, if the University put by 10 per cent. on £200 per annum at 4 per cent. compound interest, there would accrue at the expiration of thirty years a sum of £1,167, which is £51 in excess of the *conditional* cash payment offered under that option. Some of the men must die before reaching sixty years of age, and if the life-insurance element were eliminated, the moneys paid on their account would accumulate to meet unforeseen expenditure and to reduce the payments of 10 per cent. on account of each employee.

The system of pensioning by the aid of insurance companies no doubt presents a way out of the difficulty of providing for men moving from one hospital to another; but it is a system that is likely to bring more profit to insurance companies than benefits to superannuated employees.

The average amount deducted from premiums by insurance companies for management, advertising, commission to agents, etc., is from 15 per cent. to 20 per cent. and upwards, so that about one-fifth of the money paid in towards the pensions of officials is lost to them in their ultimate pension.

Hospital officials do not ask for a scheme of life insurance or for a part of the premiums of their life insurance policies to be paid for them by their employers, this they can look after for themselves. What they do need is to be protected and provided for in the event of their living beyond the period when they are able to work.

Now, the Asylums Officers' Superannuation Act, 1909 (9 Edward 7, ch. 48), is a true pension scheme, and, although small contributions of from 2 per cent. to 3 per cent. are exacted from the employee, is free from all the defects of the Federated Superannuation System. The small percentage deducted from salary is returnable to the employee who leaves the service under certain circumstances, or to his next-of-kin in the event of death before attaining the age of retirement. Those engaged in the actual care of the insane retire at fifty-five with one-fiftieth of pay and emoluments, while others retire at sixty with one-sixtieth. This is an admirable Act, and, though I contend that exacting a contribution from an employee is a mistake, the hospital employee would indeed be fortunate if the benefits thereof were extended to him.

The first thing to be done is to fix a definite and uniform rate of pensions, and it would seem impossible to do this without the aid of some central authority.

I have already suggested in a previous paper the formation of a pension fund by King Edward's Hospital Fund for London in the following way: That each hospital should pay a percentage on ordinary expenditure or on salaries and wages; that this percentage should be held by the King's Fund and all pensions paid by that body, on the recommendation of the board of management of the institution that requires a pension for its employee. The percentage would vary from year to year according to the number of persons on the pension list, the King's Fund annually assessing the rate, much in the same way as the Government assesses a figure in the estimates for Government officials. One per cent. on the expenditure of the London hospitals would probably meet all requirements. The percentage might be paid either by the hospitals to the King's Fund or deducted by the Fund from the annual grant. The chief difficulty that presents itself in this scheme is that the King's Fund does not make a grant to every hospital in London, and so would have to come to some other arrangement with those hospitals which do not receive a grant.

Another suggestion is that a central authority should require every hospital to grant a pension on a definitely arranged basis to every employee after (say) twenty years of service, on attaining sixty years of age, somewhat in the following manner: Say an employee of sixty years of age is entitled on the rate of the Superannuation Act to a pension of £300 per annum, and that his service has been given in three hospitals, it will be necessary to calculate what proportion each institution shall bear of this amount, and it might be arrived at in the following manner: Suppose that during his whole service he has earned in pay and emoluments a total of £10,000, and if the first hospital in which he served had paid him £1,500, the second £3,500, and the third £5,000, then the first hospital would pay three-twentieths of his pension, the second seven-twentieths, and the third ten-twentieths. Such a scheme as this would ensure that each hospital paid the right proportion in respect to each employee, whether his service had been wholly in one hospital or in several.

To make these schemes efficient it would be necessary for the central authority to enter into an agreement with regard to pensions when making grants, and it would probably be preferable that the indebtedness of each hospital in the matter of pensions should be paid to the central authority, and by that authority direct to the person pensioned. It would not be necessary to create an endowment fund or to hold any large amount in reserve. There need be no difficulty in regard to investments, trustees, and changing rates of interest—matters so necessary for the consideration of insurance companies and the like, and for which protective rates have to be charged. Pensions are items of ordinary expenditure. No one thinks of creating an endowment fund or forming a reserve for the payment of wages or to defray the butcher's account, and the same applies to pensions. The difficulty lies in getting some central authority to control and distribute with equity the ordinary payments of the hospitals under the head of pensions.

There seems no reason why a scheme such as mentioned above should not be extended to the provinces; it would probably be welcomed by boards of management throughout the country as relieving them of a difficulty, and providing for their old servants on a reasonable and economical basis.

Practically every hospital in the kingdom receives some grant from the King's Fund, or a Hospital Sunday, Saturday, or similar Fund, so that if a working arrangement were come to between the King's Fund and the various other funds throughout the kingdom it would be possible to assure to every hospital employee a reasonable pension on a uniform basis, and one that would probably impose but little greater burden on the hospitals of the country than the present unsatisfactory method; for every employee can now look forward to the probability, almost the certainty, of a pension if he lives long enough.

I fear it is not feasible to devise a scheme that will do justice to every person now in the service of the voluntary hospitals. The older men must stand aside and take their chance as heretofore, but in the interest of the medical charities some arrangement should be come to in regard to the younger employees and those entering hospital life.

In any scheme that may be adopted it would seem advisable to divide the employees into two classes, as in the Asylums Officers' Superannuation Act, dividing nurses and the female staff from the other employees. This method has been adopted in the pension schemes of Guy's, the London, and "Bart.'s." Women should be able to retire at a younger age than men, and the lesser rate of their remuneration renders it necessary to give them certain advantages. Besides, the great majority are not permanent employees; probably not more than 5 per cent. remain in hospital employ until of pensionable age.

It is now ten years since I read my last paper on the subject, and I must admit that I look over it with a feeling of disappointment that so little has been done since then. Progress there has been, but it has brought us little nearer a solution of the matter. It appeared at that time as though the public were becoming interested; pensions were being arranged in almost every walk of official life; but that interest seems to have waned. Lord Knutsford had recently carried his pension scheme at the London; Mr. Danvers Power, Sir Savile Crossley, and others had expressed their approval. The *Globe*, in reviewing the subject towards the end of 1902, remarked:—"There is no greater blight on human energies than the feeling that, no matter how hard or how faithfully an employee may work, his future depends upon adventitious circumstances over which he has no control."

Yet here we are to-day face to face with the problem but with the good news that King Edward's Hospital Fund for London has taken the matter up.

It is not for me to forecast the action of the King's Fund; indeed, I have felt some doubt as to whether I was justified in reading this paper lest it be thought that I attempt to dictate or discuss a matter that is *sub judice*. I trust that my action in acceding to the wish of the Council of the British Hospitals Association will be regarded merely in the light of further criticism of a subject to which I have devoted attention for many years, and as an expression of opinion of one of the oldest hospital employees in the country.

The hospital employee is surely as worthy of consideration as the asylum worker, and if the result of the King's Fund inquiry is that a pension scheme is arranged on as generous a scale as that of Guy's, "Bart.'s," and the London, a new era will indeed have opened for the hospital employee. If it is nothing more than an authoritative expression of opinion that an old servant should be pensioned on a scale not less than that of the Act of 1859 or the Asylums Officers' Superannuation Act, many a man and woman will have cause to rejoice.

### THE DISCUSSION ON MR. MICHELLI'S PAPER.

Mr. W. A. Tait, of the Royal Infirmary, Edinburgh, said that having had some experience of appointments and the removal from hospitals of officers, he was of the opinion that really good salaries ought to be paid, careful selections ought to be made, and people should not be kept a moment longer than they were really fit for work. He did not think that it was an easy matter to discuss the second portion of the paper with reference to insurance formulæ and so on. It had been very clearly pointed out that the insurance people in going about to get work must be spending money. He did not think that the pension system could really be carried out by the hospitals themselves. He was very strongly of the opinion that no hospitals themselves could promote such a scheme; it must be made a national question. As a Scotsman he did not agree with one remark in the paper, that practically every hospital in the kingdom received some grant from the King's Fund. He did not know of any grant from that Fund to any hospital in Scotland.

### The Example of the Pension Fund.

Sir Henry Burdett said they had succeeded in giving the nurses of this country absolute safety in sickness in their working days and on their retirement. The Royal National Pension Fund for Nurses had also in its title "and for hospital officials." It was established to provide for all those to whom Mr. Michelli called attention in his paper, but it was found that there were difficulties in the initial working of so large a scheme, containing so great a body of nurses, and the officials were left out. This Fund went on working resolutely for the nurses, and it was open to the hospital officials to establish some mutual system, free from the abuses and charges to which Mr. Michelli had called attention, and for the Fund to undertake to do this work for the hospitals without any charge to them and with the guarantee that the interest earned on the money entrusted to the Fund would be returned at the proper time to those who wished to retire before entering on their pension. In another connection Mr. Michelli had mentioned that Guy's Hospital had done what he (Sir Henry) thought every hospital in the country ought to do. That scheme was not complete. It applied to those members of the staff who were connected with the nursing body. All the working nurses in the hospital and all the nurses on the private staff were provided for through the Royal National Pension Fund to which Guy's Hospital was federated, and it worked out already that some nurses on the private staff were entitled to a retiring allowance of £60 a year and upwards. That would give them an idea how things could be done. If they could simplify Mr. Michelli's proposals, have a recognised time of retirement for all hospital employees, and leave the nurses to the Royal National Pension Fund for Nurses, they would do well, as it would enable them to approach their boards of management with a much better hope of success.

### A Practical Working Basis.

They could carry out Mr. Michelli's idea perfectly well by each hospital adopting the plan which Mr. Michelli suggested. Every hospital should present an estimate of the income and expenditure for the ensuing year. If they did that, all the subscribers, when they got the report, would know what increased expenditure was required and what percentage that would represent on their own subscriptions. If a letter was sent out calling attention to that he believed they would find that the increase in their revenue would be very material, because all the annual subscribers, who were the strength of the voluntary system, would say that they must do their share, as the object in view was to have higher efficiency in the hospital. To get this they should add to what they were already giving, and the committee could recommend in their report a percentage on the expenditure or the salaries for the current year, which would represent the sum required to provide the necessary money to meet the cost of the pensions to those officials who had rendered good service in the cause of the hospital. If this was what Mr. Michelli suggested should be done, then he thought the British Hospitals Association and the hospitals of the country would owe him a debt of gratitude, and that day for the first time they would have formulated a practical working basis on which this question of pensions to hospital workers could be readily taken up and provided for in a simple way by the governing body of each hospital. Meantime, the hospitals throughout the country would have Mr. Michelli's paper laid before them, and this great question of pensions, this enforcement of justice, this encouragement of the higher service, would become once and for all a great force for good, as it would benefit not only the officials but the managers of hospitals, who would realise the grave responsibility that rested upon them as the trustees of a particular hospital for the well-being of their employees. The paper was all the more forceful coming from one in the position of Mr. Michelli, whose own pension was, he knew, absolutely secure.

Sir Riley Lord said he was a good deal out of sympathy with the paper that had been read. He entirely disagreed with the principle advocated for a pension of a non-contributory character. The paper as he understood it applied very largely to the upper staff of their infirmaries, such as the house governors and so on. In his opinion these men were well able to pay something yearly towards a pension fund, and they ought to expect them to do it. They must remember that they were not dealing with Government funds. The paper referred to the Asylum Officers' Superannuation Act, but that came out of the ratepayers' pockets, which were practically inexhaustible, and he was speaking from experience as he was a member of the Asylums Committee of Newcastle Corporation. Coming to their own hospital in Newcastle, their workmen's subscriptions amounted to over £20,000 a year. The majority of these men would have no pensions until they got to be seventy years of age, and then it would only be 5s. a week. He was on the side of a pension for everyone, for the most humble labourer just as much as for the Prime Minister of England; but let them all remember that the hospitals of this country were not Government institutions. The money for their institutions came largely from the workers who got no pension until they reached the age he had named, and even they had to pay for those pensions. They had pensions for the police in Newcastle and for their Town Hall staffs, and they all paid something for these. Why,then, should not the upper staffs of the infirmaries pay something, too, for their pensions? The writer of the paper had referred to insurance companies' expenses. He thought he put these at something like from 15 to 20 per cent. He did not know where their friend got his information from, but he could put him in the way where he would find it not to be one-third of that. This question of pensions had come up before their house committee over and over again. They had done what they could for old servants, but they had not been able to act on any definite principle.

Mr. J. H. Smethurst, Chairman of Warrington Infirmary, said he must say at the outset quite frankly that as chairman of a working-class hospital he entirely disagreed with the last speaker. While the idea of a contributory pension was all right, he thought they must see to the fact that every board of management was prepared in the first place to pay an adequate salary for the work done. He had troubled a good many secretaries and boards of management in connection with hospitals for information in order that he might have the privilege to try to raise the tone and efficiency of the institution which he had the honour to represent. In almost every case from the figures that had been provided it had been a surprise to him to find that the salaries of the secretaries had been in no way in keeping with the work or with the duties that had to be performed. He had found that various organisations dealing with infirmary funds had always thought they had a perfect right to call upon the poor secretary, and to get him to work unlimited hours, and when the time came for the results to be made known the poor secretary stood behind and let all the credit be given to the honorary workers. He hoped that hospital managers would thoroughly understand this matter. If managers in some hospitals would pay their staffs better for their services there would be no need for pensions. As an employer of labour he tried to do the best he could for those under him, and he gave consideration to them on their becoming older. He thought that boards of management ought to do the same. But they did not find many hospitals had considered the matter closely, and he must say this was the case in his own hospital. He assured them, however, that as the result of their proceedings there in Newcastle he would see that more attention was given to the question. He knew quite well that when a servant got old and there came the question of retirement it was entirely a matter of sentiment with some employers as to whether they would give him £25 or £50 a year, or nothing, as they thought he ought to have saved some money. Personally he wished to thank the writer of the paper, from which he had gained some useful information, and he repeated that his hospital staff would have consideration at a very early date.

On the motion of the Chairman, a vote of thanks was passed to Mr. Michelli for his useful paper.

Mr. Michelli, in reply, said he must thank Sir Henry Burdett for the way in which he had spoken about his paper. It might not be known to everybody present that it was through Sir Henry that he had become so intimately associated with hospitals, while Sir Henry himself had done wonders for hospitals everywhere. In going to the Conference that morning, Sir Henry Burdett had attacked him in every way upon his paper, so that, when he got there and heard what Sir Henry had to say at the Conference, it was doubly agreeable to him. His blessing on the pension scheme would go a very long way throughout the country. He (Mr. Michelli) felt very much honoured by the criticism of Sir Riley Lord. It was a great privilege to have a paper so adversely criticised by so able a critic, and by one who was so closely associated with one of the greatest insurance companies in the world. Though Sir Riley Lord had hit him very hard, he felt that he had every sympathy with the employee, and that it was his desire to get the best class of men and women into the hospitals of the kingdom. Mr. Smethurst was the practical chairman of one of the leading hospitals in the country, and it was a gratification to know that he intended taking back to his place something that had been said both in the paper and in the discussion. They who lived in London were inclined to get somewht cramped, and to think there was nothing like London, but when he visited centres like Newcastle, he realised at once the earnest work of those who sat on the boards of management and controlled the affairs of the hospital. After all, a paper was no good unless it was able to stand some knocks, as he hoped had been the case with his paper.

Mr. W. Straker, corresponding secretary of the Northumberland Miners' Association, and a member of the House Committee, Newcastle Royal Victoria Infirmary, who was to read a paper on "Proposed Amendment of the Truck Acts, 1831 to 1896, and How it may Affect the Voluntary Hospitals," said:

The Amending Bill dealing with this matter is now dropped, and the question did arise as to whether it would be worth while proceeding with this paper in that case, but I have come to the conclusion that it does not alter our attitude to the proposal in any way whatever. We all know, those who take an interest in these things, that private members do not introduce measures into the House of Commons with any hope of having them passed through. They introduce them based on the findings of a Commission with the object of commencing as far as possible the Government measure that always follows such a Commission. Therefore, while the Amending Bill is dropped we know that the promoters have got an assurance that a measure is to be brought in. That Government measure will be based on the majority report rather than on the minority report, but that will not hinder the promoters of this measure from seeking to secure their object in another way, and that will be by seeking amendments for the Government measure when it comes before the committee of the House. Our attitude, then, towards the proposal must be just as vigilant as if it were being proceeded with. After I have read my paper I understand it is intended to move a resolution, and I hope that, though there may not be much discussion on the paper, that the British Hospitals Association will continue to watch this matter as they have been doing for some time past.

